# Supplementary material for: Differential DNA methylation in blood as potential mediator of the association between ambient PM2.5 and cerebrospinal fluid biomarkers of Alzheimer’s disease among a cognitively normal population-based cohort
Source: Mol Psychiatry. 2026 Jun 10;31(10):5811–22. doi: 10.1038/s41380-026-03662-9 (PMC13358818; doi:10.1038/s41380-026-03662-9)
Supplement: Supplementary file 1 [file 41380_2026_3662_MOESM1_ESM.docx]

**Figure S1**. Directed acyclic graph illustrating the confounding structure in the analysis. The diagram depicts the associations between (A) PM_2.5_ (exposure) and the CSF Aβ_42_ level (outcome), (B) PM_2.5_ (exposure) and the DNA methylation (mediator), and (C) DNA methylation (mediator) and the CSF Aβ_42_ level (outcome). Note: PM_2.5_, fine particulate matter; Aβ_42_, beta-amyloid 42; CSF, cerebrospinal fluid; BMI, body mass index; SES, socioeconomic status. Details about covariates are provided in the Methods section.

**Figure S2**. Filtering flow chart outlining the process for data preparation and filtering for each of the five high-dimensional mediation methods used in the analysis.

**Figure S3.** Distribution of (A) 1-year ambient PM_2.5_ exposure concentration, (B) CSF Aβ_42_ concentrations, and (C) z-scores standardized Aβ_42_ levels among the study population (n=536).

**Figure S4.** QQ plots for the “one-at-a-time” (A) mediator models and (B) outcome models for 661,869 CpG sites tested for the association between PM_2.5_ exposure and CSF Aβ_42_ levels. Lambda denotes the inflation factor. Both models were adjusted for covariates: sex, age, race/ethnicity, educational attainment, BMI, ADI, three principal components of neighborhood deprivation, and proportions of blood cells. (C) Manhattan plot showing the maximum of p-value for each CpG from the mediator and outcome models. The red line indicated the raw p-value threshold of 0.05. CpGs with p<0.05 for both exposure-mediator and mediator-outcome associations (n=1,967) are colored in light blue, with 898 of them demonstrating negative indirect effects further highlighted in dark blue.

**Figure S5.** Distribution of (A) 1-year ambient PM_2.5_ exposure concentration, (B) CSF Aβ_42_ concentrations, and (C) z-score standardized Aβ_42_ levels among White participants (n=458).

**Figure S6.** Subgroup analysis among White participants (N=458). Causal mediation analysis for the association between PM_2.5_ (exposure) and AD CSF biomarker Aβ_42_ concentrations (outcome, standardized as z-scores) using noteworthy CpG sites identified through high-dimensional mediation analysis methods. This figure presents estimates for natural indirect effect (NIE), natural direct effect (NDE), total effect (TE), and proportion mediated (PM) for selected CpG sites, assessed individually. Effect estimates represent the changes in Aβ_42_ z-score concentrations per 1ug/m^3^ increase in PM_2.5_ exposure. Significant indirect effects are marked with (*), while no significant direct or total effects were observed.

**Figure S7.** Correlation heatmap for the 9 CpG sites identified as noteworthy mediators. This heatmap displays Pearson correlation coefficients for each pair of CpG sites. Significant correlations (p-value <0.05) are indicated with black estimates, while non-significant correlations are shown in grey. For significant correlations, red circle represents negative coefficients and blue circle represents positive coefficients. The size of the circle corresponds to the magnitude of the correlation coefficient.

**Table S1.** Baseline descriptive characteristics, PM_2.5_ exposure and AD CSF biomarker outcomes for the subgroup analysis among White participants.

| **Characteristics** | **Total (n=458)** |
| --- | --- |
| **Age (y)** |  |
| Mean (SD) | 62.1 (6.9) |
| Median (min, max) | 62.0 (45.0, 76.0) |
| **Gender** |  |
| Female | 317 (69.2%) |
| Male | 141 (30.7%) |
| **BMI (kg/m^2^)** |  |
| Mean (SD) | 25.3 (3.7) |
| Median (min, max) | 24.9 (16.8, 38.4) |
| **Race** |  |
| White | 458 (100.0%) |
| **Hispanic ethnicity** |  |
| Yes | 11 (2.4%) |
| No | 447 (97.6%) |
| **Education** |  |
| Less than college | 66 (14.4%) |
| College | 197 (43.0%) |
| Master or higher | 195 (42.6%) |
| **Area Deprivation Index** |  |
| Mean (SD) | 27.3 (19.1) |
| Median (min, max) | 23.0 (1.0, 93.0) |
| **Air pollution concentration** |  |
| **1-y ambient PM_2.5_ (μg/m^3^)** |  |
| Mean (SD) | 9.51 (0.76) |
| Median (min, max) | 9.52 (6.99, 13.21) |
| IQR | 0.80 |
| **AD CSF concentration and (+) cutoff** |  |
| **Aβ_42_ (pg/mL)** |  |
| Mean (SD) | 1216.3 (374.7) |
| Median (min, max) | 1230.5 (200.0, 1700.0) |
| IQR | 640.8 |
| **Z-score Aβ_42_** |  |
| Mean (SD) | 0.00 (1.00) |
| Median (min, max) | 0.34 (-2.71, 1.29) |
| IQR | 1.71 |

Note: EHBS, Emory Healthy Brain Study; PM_2.5_, fine particulate matter; Aβ_42_, beta-amyloid 42; AD, Alzheimer’s disease; CSF, cerebrospinal fluid; BMI, body mass index; IQR, interquartile range; max, maximum; min, minimum.

**Table S2.** Baseline descriptive characteristics, PM_2.5_ exposure and AD CSF biomarker outcomes for the Casey et al. 2024 cohort

| **Characteristics** | **Total (n=1,113)** |
| --- | --- |
| **Age (y)** |  |
| Mean (SD) | 61.7 (6.7) |
| Median (min, max) | 62.0 (45.0, 77.0) |
| **Gender** |  |
| Female | 775 (69.6%) |
| Male | 338 (30.4%) |
| **BMI (kg/m^2^)** |  |
| Mean (SD) | 25.5 (3.6) |
| Median (min, max) | 25.3 (16.8, 38.4) |
| **Race** |  |
| White | 943 (84.7%) |
| Black/African American | 121 (10.9%) |
| Other | 49 (4.4%) |
| **Hispanic ethnicity** |  |
| Yes | 32 (2.9%) |
| No | 1,081 (97.1%) |
| **Education** |  |
| Less than college | 147 (13.2%) |
| College | 478 (42.9%) |
| Master or higher | 488 (43.8%) |
| **Area Deprivation Index** |  |
| Mean (SD) | 29.4 (20.1) |
| Median (min, max) | 25.0 (1.0, 93.0) |
| **Air pollution concentration** |  |
| **1-y ambient PM_2.5_ (μg/m^3^)** |  |
| Mean (SD) | 9.52 (0.76) |
| Median (min, max) | 9.52 (5.63, 13.20) |
| IQR | 0.84 |
| **Aβ_42_ CSF concentration** |  |
| **Aβ_42_ (pg/mL)** |  |
| Mean (SD) | 1200.0 (382.0) |
| Median (min, max) | 1210.0 (200.0, 1700.0) |
| IQR | 692.3 |
| **Z-score Aβ_42_** |  |
| Mean (SD) | 0.00 (1.00) |
| Median (min, max) | 0.02 (-2.62, 1.30) |
| IQR | 1.81 |

Note: EHBS, Emory Healthy Brain Study; PM_2.5_, fine particulate matter; Aβ_42_, beta-amyloid 42; AD, Alzheimer’s disease; CSF, cerebrospinal fluid; BMI, body mass index; IQR, interquartile range; max, maximum; min, minimum.

**Table S3.** Associations between per 1 μg/m^3^ increase in ambient PM_2.5_ exposure and CSF Aβ_42_ levels in the main analysis cohort and sensitivity analysis cohorts.

| **Cohort** | **N** | **Beta** | **95% CI** | **P-value** |
| --- | --- | --- | --- | --- |
| Main analysis cohort | 536 | -0.074 | (-0.190, 0.042) | 0.213 |
| Sensitivity analysis cohort among White participants | 458 | -0.086 | (-0.210, 0.039) | 0.177 |
| Casey et al. 2024 cohort | 1113 | -0.102 | (-0.179, -0.024) | 0.010 |

Note: Associations were estimated using multiple linear regression adjusted by sex, age, race/ethnicity, educational attainment, BMI, Area Deprivation Index, and three principal components of neighborhood deprivation. Casey et al. 2024 cohort (N=1113) includes the Emory Healthy Brain Study participants used to assess the total association between PM_2.5_ exposure and CSF biomarkers of Alzheimer’s disease. The main analysis cohort (N=536) in this study is a subset of the Casey et al. (2024) cohort with DNA methylation data. A sensitivity analysis cohort was restricted to White participants (N=458) with DNA methylation data. The outcome, Aβ_42_ concentration, was standardized as z-score.

**Table S4. (In additional file)** Estimated mediation effects and the raw joint significance test p-values for 133 active CpG sites identified by at least one high-dimensional mediation analysis methods (DACT, HIMA1, HIMA2, HDMA, MedFix) for the associations between PM_2.5_ exposure and AD CSF biomarker Aβ_42_ concentrations.

**Table S5. (In additional file)** BECon Blood-Brain Epigenetic Concordance for the 9 noteworthy CpG sties identified through high-dimensional mediation analyses.

**Table S6. (In additional file)** Blood-Brain Epigenetic Concordance for the 9 noteworthy CpG sties identified through high-dimensional mediation analyses using the Gene Expression Omnibus Database [Accession code GSE111165].

**Table S7.** **(In additional file)** GO terms that were nominally significant (p<0.05) for the 898 CpG sites filtered from the first step.

**Table S8.** **(In additional file)** Top 20 KEGG pathways based on the 898 CpG sites filtered from the first step.
